# Risankizumab shows high efficacy and maintenance in improvement of response until week 52

**DOI:** 10.1111/dth.15378

**Published:** 2022-02-28

**Authors:** Luca Mastorino, Sara Susca, Matteo Megna, Niccolò Siliquini, Pietro Quaglino, Michela Ortoncelli, Gianluca Avallone, Marco Rubatto, Gabriella Fabbrocini, Paolo Dapavo, Simone Ribero

**Affiliations:** ^1^ Department of Medical Sciences, Section of Dermatology University of Turin Torino Italy; ^2^ Section of Dermatology, Department of Clinical Medicine and Surgery University of Naples Federico II Naples Italy

**Keywords:** biological therapy, BMI, psoriasis, psoriatic arthritis, risankizumab, smoking habits

## Abstract

Risankizumab has been recently approved for moderate‐to‐severe plaque psoriasis; however, real‐life studies are scarce. Analysis of possible predictor factors of treatment response are limited to body mass index (BMI) and previous biologic experience. Our objectives were to evaluate the effectiveness and safety of Risankizumab and to investigate on possible predictor factors response. We retrospectively analyzed 166 patients from two centers in Italy who undergone Risankizumab for psoriasis. The proportion of patients achieving a 100%, 90%, 75% of improvement in Psoriasis Area Severity Index (PASI) and PASI <3 were collected at weeks 16, 28, 40, and 52. Study population was analyzed in subgroups to investigate possible predictors of response to Risankizumab since week 40. At the time of analysis 165, 103, 30, and 11 patients had completed 16, 28, 40, and 52 weeks of treatment, respectively. The mean PASI score decreased from 12.5 ± 5.1 at baseline to 1.9 ± 2.4 at week 16. Similar reductions were observed when considering PASI <3, PASI 75, PASI 90, and PASI 100. Previous biologics failure, different smoking habits, obesity, and joint involvement resulted in a lower response to risankizumab. In particular, significant differences in mean PASI at any time‐points was observed between psoriatic arthritis (PSA) and non‐PSA patients: 2.7 versus 1.7 (*p* = 0.036), 1.9 versus 0.4 (*p* = 0.006), and 4.1 versus 0.5 (*p* = 0.016) at 16, 28, and 40 weeks, respectively. No difference in response to risankizumab occurred in the case of involvement of difficult‐to‐treat areas. In this population, Risankizumab was effective and safe. Smoking habits, joint involvement, obese status, and previous biologic experience may negatively affect treatment response, while difficult body sites involvement have minor impact.

## 1 INTRODUCTION

Risankizumab is the latest biologic treatment approved by Food and Drug Administration and European Medical Agency for moderate‐to‐severe plaque psoriasis.^1^, ^2^ This new biologic, a humanized IgG1 monoclonal antibody, targets the p19 subunit of IL‐23.^3^

High levels of efficacy and safety have been demonstrated in different clinical trials (UltIMMA‐1 and ‐2, IMMerge, IMMvent, SustaIMM).^4^, ^5^, ^6^, ^7^ In these studies, a great percentage of patients reached PASI 90 (>70%) and complete remission at 16 weeks. This result was supported by real‐world evidence available in the literature since now.^2^, ^3^, ^8^, ^9^, ^10^ Real‐life studies were scarce and with small sample size and short follow‐up, due to the novelty of the drug, most of the studies are limited to the 16th week and in the following weeks the sample drops significantly.^2^, ^3^, ^10^

The analysis of possible response factors to treatment with risankizumab is limited in clinical trials and real‐life studies to body mass index (BMI) and the use of previous biologic therapy.^2^, ^9^ Real‐world data show good responses in difficult‐to‐treat areas such as the nails and the palm‐plantar area.^3^, ^10^ Although new systemic treatments and recent literature reviews have reduced the weight of these difficult locations (scalp, nails, folds, genital sites, and palms and soles of the feet) in the course of psoriatic disease, these sites may still show a slower response to the new biologics.^3^, ^10^, ^11^ Lifestyle habits, such as smoking, negatively impact the course of psoriasis through the production of free radicals that activate the tumor necrosis factor (TNF)‐alfa and Janus kinase (JAK) pathways.^12^, ^13^ No data on the impact of smoking habits on risankizumab response are available in literature.

The presence of joint involvement, psoriatic arthritis (PSA), shares the same inflammatory pathways as the cutaneous form, however, the greater resistance to treatment and the deeper systemic involvement suggests the role of further molecular and genetic factors, not yet well defined. The impact on the quality of life of PSA is often greater than its skin counterpart and therapeutic options lead to more modest results.^14^

The high impact on quality of life of PsA and involvement of difficult sites justifies the prescription of biologic drugs even in patients with a limited extent of disease [i.e., low Psoriasis Area Severity Index (PASI)].^3^, ^14^

The purpose of our study was to assess the effectiveness and safety of risankizumab and possible prognostic factors as the use of previous biological therapies, obese status, the involvement of difficult sites, the habit of smoking, and joint involvement in real life setting.

## 2 METHODS

This was a retrospective multicenter study with the aim of analyzing data and possible demographic markers of prognostic response in adult patients treated with Risankizumab for moderate‐to‐severe psoriasis in two tertiary centers in Italy, the Dermatology Clinic of the University of Torino and the Dermatology Clinic of the University of Naples Fedeico II. We included all patients who received at least one dose of Risankizumab 150 mg administered subcutaneously. The cutoff date of our analysis was 31 August 2021. The study was conducted under the 1964 Declaration of Helsinki and all subsequent amendments, and all patients provided informed consent. At the initial visit, demographic data (age, sex, height, BMI), and data on comorbidities (particularly cardiovascular disease and diabetes mellitus status), medical history, smoking status, presence of PsA, and previous systemic and biologic therapy were obtained. In addition, age of psoriasis onset, bio‐naïve status, and psoriasis type was recorded.

Data on PASI and adverse events were collected at patient visits at weeks 16, 28, 40, 52 when Risankizumab was administered. We focused on absolute PASI, PASI 100, PASI 90, PASI 75 and PASI <3. We categorized our patient according to BMI (≥30 or <30 id est obese vs. non‐obese), smoking status (smoker, non‐smoker, ex‐smoker), the presence of joint involvement, previous biologic therapy, and difficult‐to‐treat areas involvement (we considered difficult site palm and plantar area, scalp, genital, nails and fold localization).

## 3 STATISTICAL ANALYSIS

For this analysis, epidemiological data (i.e., demographic and disease characteristics and medical history), disease severity (PASI), BMI, cardiovascular comorbidities, diabetes mellitus status, and previous treatments were summarized using descriptive statistics. Descriptive statistics were used to evaluate the data set according to the number of patients and their percentage proportion in the groups related to the categorical variables; mean and standard deviation (SD) were used for continuous variables. Inferential statistics were performed up to week 40 due to low sample sizes at 52 weeks. The categorical variables were analyzed using the chi‐square test and Exact Fisher's Test where needed, while the continuous variables were tested using the Shapiro–Wilk test to investigate the normality of the distribution, then the dichotomous normal distributions were compared using the Student's *t* test, non‐normal distributions were tested using the Mann–Whitney *U* test if dichotomous. Kruskal‐Wallis test were used to compare more than two distributions.

## 4 RESULTS

Since September 2020, a total of 166 patients were treated at the two centers with risankizumab for moderate‐to‐severe psoriasis and enrolled in this study. Of these 166 patients, 66% were male (*n* = 108), the mean age of the patients was 65.1 (SD 18.6), the mean age of onset of psoriasis was 32.1 (ds 16.6). At baseline, the mean BMI was 27 (SD 6) and 36 (22%) patients were obese (BMI > 30). 66% of patients reported at least one comorbidity, cardiovascular and metabolic diseases were present in 28% and 8% of patients, respectively. Regarding concomitant infectious diseases, 5 patients had a history of HCV, 2 of HBV, 1 of HIV, at a young age one patient had viral encephalitis and another reported a history of tuberculosis 43 years previously, 1 patient tested positive for Quantiferon‐TB screening without subsequent prophylactic therapy. During the weeks of follow‐up, there was no reactivation of the disease and no further infection. Of note, three patients had experienced low‐grade bladder carcinoma more than 5 years previously and 1 patient had experienced osteosarcoma with lung metastases more than 20 years previously. Thirty‐eight percent of patients were active smokers, 23% reported quitting smoking and 39% had never smoked. Twenty‐one (13%) patients also suffered from PsA and 72 (43%) patients had at least one complicated site involved (i.e., nails, folds, palms and soles, and genitals).

Methotrexate (59%) was the most common previous treatment, followed by cyclosporine (49%), acitretin (21%), phototherapy (16%), apremilast (5%) and dimethyl fumarate (1%). Bio‐naive and bio‐experienced patients accounted for 42% and 58%, respectively. Among the bio‐experienced, ustekinumab represented the most common one (25%), followed by adalimumab (21%) ixekizumab (20%), secukinumab (16%), etanercept in 7%, infliximab in 7%, brodalumab in 6%, guselkumab in 5%, certolizumab in 2%, %, and tildrakizumab in 0.6%. Twelve patients (7%) took risankizumab after failing at least 4 biological treatments and were identified as multi‐failure.

Baseline features of the study population are depicted in Table 1. The mean PASI score at baseline was 12.5 SD 5.4) with the highest score being 30 (Table 1).

**TABLE 1:** Baseline characteristics of the population in study

Number of patients	166
Male	108 (66%)
Age	65.1 SD18.6
Age at psoriasis diagnosis	32.1 SD 16.6
BMI	27 SD 6
Obese (BMI ≥ 30)	36 (22%)
PsA	23 (14%)
Difficult‐site involvement	119 (73%)
Comorbidity	108 (66%)
CV disease	63 (28%)
DM II	14 (8%)
Smokers	63 (38%)
Ex‐Smokers	40 (23%)
Non‐Smokers	62 (39%)
Bio‐Naive	69 (42%)
*Previous biologic treatments*	
Adalimumab	35 (21%)
Etanercept	12 (7%)
Infliximab	12 (7%)
Certolizumab	4 (2%)
Ustekinumab	42 (25%)
Secukinumab	27 (16%)
Ixekizumab	34 (20%)
Brodalumab	10 (6%)
Guselkumab	9 (5%)
Tildrakizumab	1 (0.6%)
Multifailure for biologics (≥4)	12 (7%)
*Previous non biologic systemic treatment*	
Phototherapy	27 (16%)
Methotrexate	98 (59%)
Cyclosporine	82 (49%)
Acitretin	35 (21%)
Apremilast	8 (5%)
*Dimethyl fumarate*	2 (1%)
Baseline PASI	12.5 SD 5.1

Abbreviations: BMI, body mass index; CV, cardiovascular; DM II, diabetes mellitus II; PASI, Psoriasis Area Severity Index; PSA, psoriatic arthritis; SD, standard deviation.

## 5 RISANKIZUMAB TREATMENT

All patients received at least one dose of risankizumab. At the time of analysis 165 (99.9%), 10 3(62%), 30 (18%) and 11 (7%) patients had completed 16 weeks, 28 weeks, 40 weeks, and 52 weeks of treatment, respectively.

**FIGURE 1:**
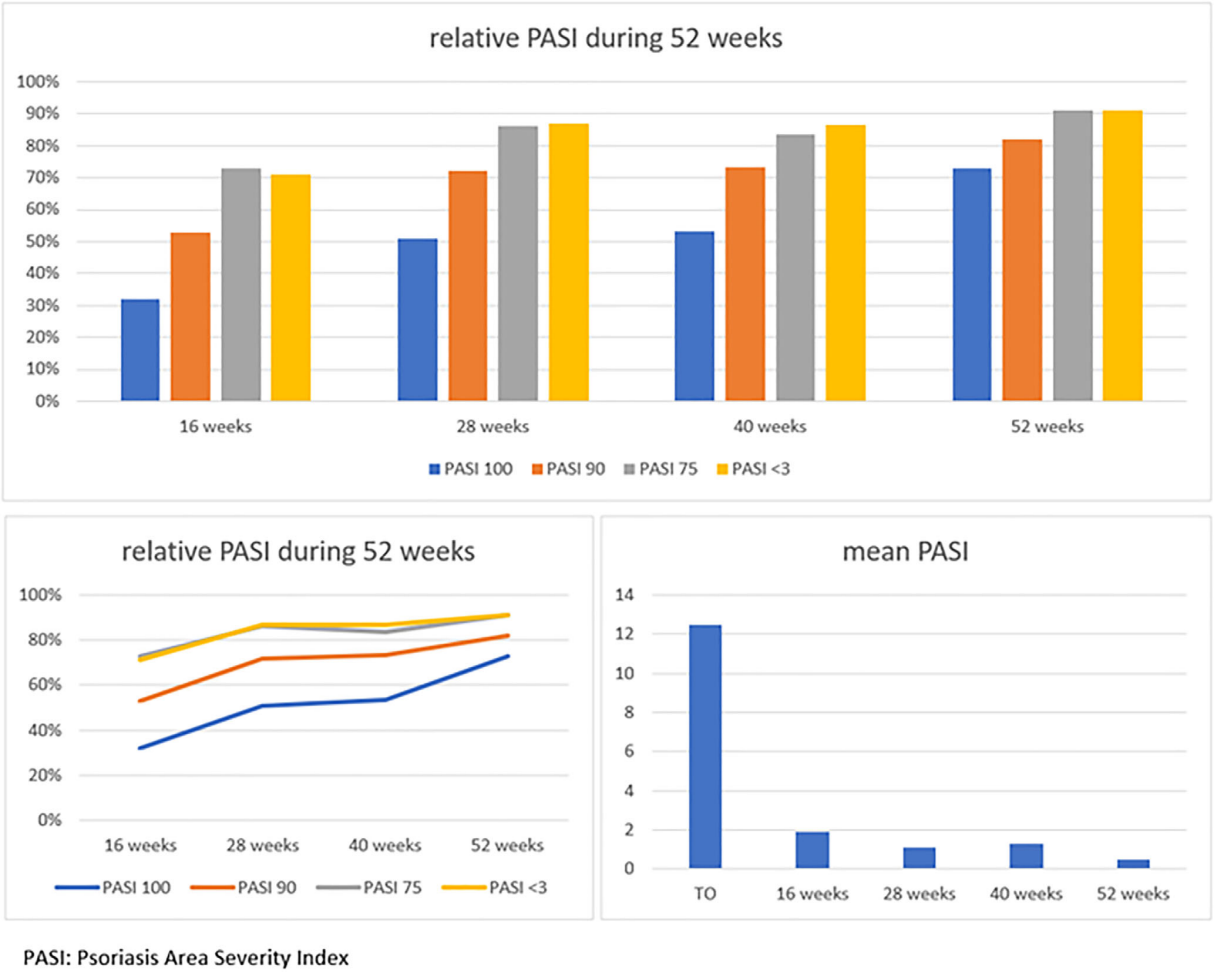
Graphic reduction in mPASI and in the achievement of PASI 100, 90, 75, and <3 in general population during weeks 16, 28, 40, and 52

The mean PASI score significantly decreased from 12.5 (SD 5.1) at baseline, to 1.9 (SD 2.4) at week 16. Further reductions were observed at 28, 40, and 52 weeks with a mean PASI score of 1.1 (2 SD), 1.3 (2.7 SD), and 0.5 (0.5 SD), respectively (*p* = 0.000). Similar reductions were observed when considering PASI <3, PASI 75, PASI 90, and PASI 100. The absolute PASI <3 score was achieved by 71% of patients on treatment at week 16, and by 87%, 87%, and 91% of patients at weeks 28, 40, and 52, respectively. PASI 75 was achieved by 73% of patients at week 16 and subsequently by 86%, 83%, and 91% at weeks 28, 40, and 52. PASI 90 was achieved by 53% of patients at week 16, and by 72%, 73%, and 82% at weeks 28, 40, and 52, respectively. Finally, PASI 100 was observed in 32% of patients on treatment at week 16, and in 51%, 53%, and 73% at weeks 28, 40, and 52 (Table 2) (Figure 1).

**TABLE 2:** Reduction in mPASI and in the achievement of PASI 100, 90, 75, and <3 in general population during weeks 16, 28, 40, and 52

		16 weeks	28 weeks	40 weeks	52 weeks
mPASI	12.5 (SD 5.1)	1.9 (SD 2.4)	1.1 (SD 2)	1.3 (SD 2.7)	0.5 (SD 0.5)
PASI 100		32%	51%	53%	73%
PASI 90		53%	72%	73%	82%
PASI 75		73%	86%	83%	91%
PASI <3		71%	87%	87%	91%

Abbreviation: mPASI, mean Psoriasis Area Severity Index.

Previous biological therapy failure showed impact on the response to risankizumab in our analysis. Particularly, the mean PASI in bio‐naive and bio‐experienced patients at 16 weeks was 1.3 and 2.3 (*p* = 0.047), in the same time frame 65% and 44% had reached PASI 90, (*p* = 0. 007), and 83% and 66% reached PASI 75 (*p* = 0.020), respectively. At 28 weeks 86% and 62% reached PASI 90 (*p* = 0.008) and 95% and 80% reached absolute PASI <3 (*p* = 0.0043), respectively, in bio‐naïve and bio‐experienced patients. Statistically significant differences were also detected at week 40 in the achievement of PASI 90; at this time 100% of bio‐naïve patients reached the outcome versus 64% in bio‐experienced (*p* = 0.046).

The involvement of difficult sites did not result in significant differences at any time points analyzed, except for the achievement of PASI 90 at week 16. The mean PASI in patients with or without difficult sites involvement at 16 weeks was 1.8 versus 1.6 (*p* = 0.101); at the same time point 28% and 41% reached PASI 100, (*p* = 0.130), 47% and 69%, reached PASI 90 (*p* = 0.012), 71% and 80% reached PASI 75 (*p* = 0.225) and 67% and 80% reached absolute PASI <3 (*p* = 0.109), respectively.

Regarding BMI status, significant differences were observed in obese patients (BMI ≥ 30) compared to non‐obese. Mean PASI at 28 weeks was 1.7 in obese patients and 0.9 in non‐obese (*p* = 0.006). PASI 100 at weeks 16 and 28 was reached by 22% and 13% in obese patients and by 58% and 68% in non‐obese patients, respectively (*p* = 0.007 at week 16 and *p* = 0.007 at week 28). At 40 weeks significant differences were observed in the achievement of PASI <3: 63% in obese patients and 95% in non‐obese patients (*p* = 0.019).

Our analysis showed that smoking habits impact on PASI endpoints less significantly and less consistently than obesity. Differences were observed in the mean PASI at 40 weeks between smokers, non‐smokers and ex‐smokers: 2.7, 1, and 0.5, respectively (*p* = 0.026). A significant difference was, also, observed in the achievement of PASI 100 at 40 weeks: 30% of smokers, 38% of ex‐smokers and 83% of non‐smokers reach this outcome (*p* = 0.029).

In our study, PsA presence seems to deeply influence risankizumab outcome. Significant differences in mean PASI at any time‐points was observed between PSA and non‐PSA patients: 2.7 versus 1.7 (*p* = 0.036), 1.9 versus 0.4 (*p* = 0.006) and 4.1 versus 0.5 (*p* = 0.016) at 16, 28, 40 weeks respectively. In addition, further differences were described in the achievement of PASI 100 at 28 and 40 weeks, PASI 75 at 16 and 40 week, PASI 90 at 28 week and 40 week, and absolute PASI <3 at 28 and 40 week: 25% versus 56% (*p* = 0.024), 0% versus 67% (*p* = 0.003), 50% versus 77% (*p* = 0.008), 50% versus 92% (*p* = 0.014), 50% versus 76% (*p* = 0.037), 17% versus 88% (0.000), 69% versus 91% (*p* = 0.016) and 50% versus 95% (*p* = 0.003), respectively.

**FIGURE 2:**
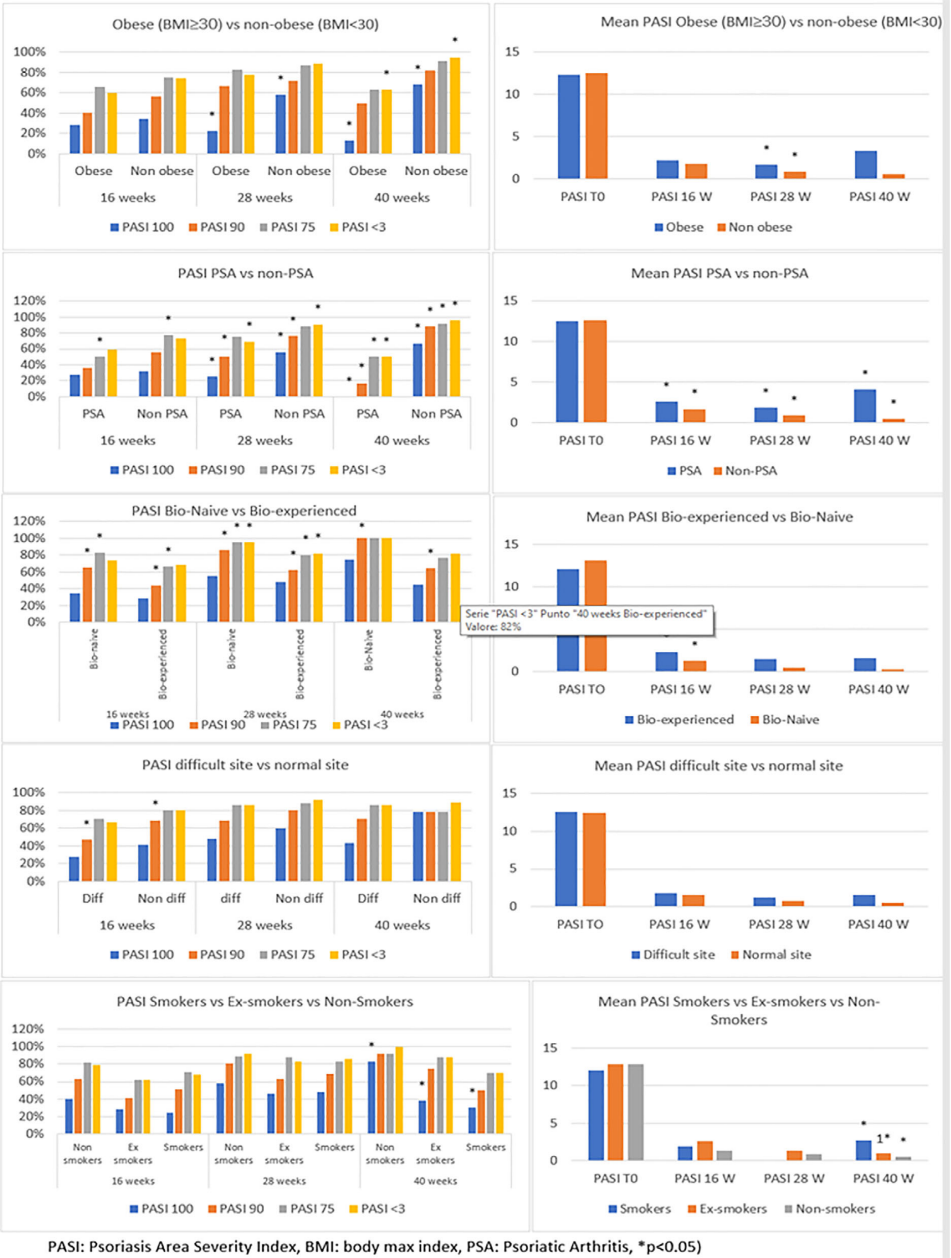
Graphic reduction in mPASI and in the achievement of PASI 100, 90, 75, and <3 in general population during weeks 16, 28, and 40 according to previous biologic status, BMI status, difficult sites involvement, smoking habits and joint involvement

Results were summarized in Table 3 and Figure 2. No significant differences were showed in the mean PASI at baseline for each subgroups evaluated.

**TABLE 3:** Comparative reduction in mPASI and in the achievement of PASI 100, 90, 75, and <3 in the subpopulation analyzed (obese vs. non‐obese, PSA vs. non‐PSA, previous biologic status, difficult‐site involvement and smoking habits) during weeks 16, 28, and 40

	BMI ≥ 30		BMI < 30		*p*‐value	PSA		Non PSA		*p*‐value	Bio‐Experienced		Bio‐naive		*p*‐value	Difficult‐site		Non difficult‐site		*p*‐value	Smokers		Ex‐Smokers		Non‐Smokers		*p*‐value
mPASI T0	12.3		12.5		0.259	12.5		12.6		0.791	12.1		13.1		0.106	12.6		12.5		0.855	12.0		12.9		12.8		0.861
mPASI 16 W	2.2		1.8		0.101	2.7		1.7		0.036	2.3		1.3		**0.047**	1.8		1.6		0.101	1.9		2.6		1.4		0.050
PASI 100 16 W	8/35	28%	43/129	34%	0.258	6/22	27%	45/142	32%	0.677	27/95	28%	24/69	35%	0.385	33/119	28%	18/45	41%	0.130	15/63	24%	11/39	28%	25/62	40%	0.124
PASI 90 16 W	12/35	40%	73/129	56%	0.088	8/22	36%	79/142	56%	0.092	42/95	44%	45/69	65%	**0.007**	56/119	47%	31/45	69%	**0.012**	32/63	51%	16/39	41%	39/62	63%	0.090
PASI 75 16 W	24/35	66%	96/129	75%	0.505	11/22	50%	109/142	77%	**0.008**	63/95	66%	57/69	83%	**0.020**	84/119	71%	36/45	80%	0.225	45/63	71%	24/39	62%	51/62	82%	0.067
PASI <3 16 W	21/35	60%	96/129	74%	0.122	13/22	59%	103/142	73%	0.197	65/95	68%	51/69	74%	0.445	80/119	67%	36/45	80%	0.109	43/63	68%	24/39	62%	49/62	79%	0.146
mPASI 28 W	1.7		0.9		**0.006**	1.9		0.9		0 0.006	1.5		0.5		0.175	1.2		0.8		0.260	1.1		1.3		0.9		0.497
PASI 100 28 W	4/18	22%	48/84	58%	**0.007**	4/16	25%	48/86	56%	**0.024**	29/60	48%	23/42	55%	0.523	37/77	48%	15/25	60%	0.299	20/42	48%	11/24	46%	21/36	58%	0.542
PASI 90 28 W	12/18	67%	61/84	72%	0.611	8/16	50%	65/86	76%	**0.037**	37/60	62%	36/42	86%	**0.008**	53/77	69%	20/25	80%	0.282	29/42	69%	15/24	63%	29/36	81%	0.282
PASI 75 28 W	15/18	83%	73/84	87%	0.689	12/16	75%	76/86	88%	0.154	48/60	80%	40/42	95%	**0.028**	66/77	86%	22/25	88%	0.773	35/42	83%	21/24	88%	32/36	89%	0.761
PASI <3 28 W	14/18	78%	75/84	89%	0.184	11/16	69%	78/86	91%	**0.016**	49/60	82%	40/42	95%	**0.043**	66/77	86%	23/25	92%	0.413	36/42	86%	20/24	83%	33/36	92%	0.591
mPASI 40 W	3.3		0.6		0.059	4.1		0.5		0.016	1.6		0.3		0.114	1.6		0.5		0.184	2.7		1		0.5		**0.029ª**
PASI 100 40 W	1/8	13%	15/22	68%	**0.007**	0/6	0%	16/24	67%	**0.003**	10/22	45%	6/8	75%	0.151	9/21	43%	7/9	78%	0.079	3/10	30%	3/8	38%	10/12	83%	**0.026ª**
PASI 90 40 W	4/8	50%	18/22	82%	0.081	1/6	17%	21/24	88%	**0.000**	14/22	64%	8/8	100%	**0.046**	15/21	71%	7/9	78%	0.719	5/10	50%	6/8	75%	11/12	92%	0.088
PASI 75 40 W	5/8	63%	20/22	91%	0.065	3/6	50%	22/24	92%	**0.014**	17/22	77%	8/8	100%	0.140	18/21	86%	7/9	78%	0.593	7/10	70%	7/8	88%	11/12	92%	0.372
PASI <3 40 W	5/8	63%	21/22	95%	**0.019**	3/6	50%	23/24	96%	**0.003**	18/22	82%	8/8	100%	0.195	18/21	86%	8/9	89%	0.815	7/10	70%	7/8	88%	12/12	100%	0.119

Abbreviations: BMI, body max index; mPASI, mean Psoriasis area severity index. ^a^ Smokers versus non‐smokers. Bold values are p<0.05.

During the study period, only 7 adverse events were observed, 1 (0.6%) of these caused treatment interruptions: a case of peri‐malleolar edema. A case of death occurred during study treatment for causes not related to the treatment. The most frequent adverse event was upper respiratory tract infection that was experienced by two patients. In total only 8 patients (4.8%) interrupted the treatment; the majority (6/8 75%) due to primary inefficacy. Cyclosporine was the predilect treatment of switch (3 patients), followed by brodalumab and certolizumab pegol (2 and 1 patients, respectively) (Table 4).

**TABLE 4:** side effects, cause of interruption and treatment of switch

Adverse events	N° (% on total population)
Upper respiratory tract infections	2 (1.2%)
Cefalea	1 (0.6%)
Arthralgya	1 (0.6%)
Peri‐malleolar edema	1 (0.6%)
Diarrhea	1 (0.6%)
Death (not correlated to the treatment)	1 (0.6%)
Total	7 (4.2%)

Cause of interruption	N° (% on total population)
Primary inefficacy	6 (3.6%)
Peri‐malleolar edema	1 (0.6%)
Total	7 (4.2%)

Treatment of switch	N° (% on total population)
Cyclosporine	3 (1.8%)
Brodalumab	2 (1.2%)
Certolizumab Pegol	1 (0.6%)
None	1 (0.6%)
Total	7 (4.2%)

## 6 DISCUSSION

As far as we know, our retrospective real‐life multicenter study is one of the largest reported in the literature.^2^, ^3^, ^8^, ^9^, ^10^ Unfortunately, the numbers of patients at weeks 40 and 52 were low, which is one of the possible limitations of a study like ours mostly due to the recent availability of the drug.

Risankizumab proved to be effective and safe in the treatment of moderate–severe psoriasis. The reduction of the mean PASI in our population was consistent as early as 16 weeks and showed a tendency for further reduction at subsequent endpoints. The mean PASI score decreased from 12.5 (SD 5.1) at baseline to 1.9 (SD 2.4) at week 16 which is similar to the mean PASI score reported in the Czech study by Gkalpakiotis et al. on 94 patients and an Italian study by Megna et al. on 8 patients, 1.7 (SD 2.3) and 3.3 (SD 1.7), respectively.^2^, ^10^ We observed a similar reduction in the achievement of PASI 100, PASI 90, PASI 75, and PASI <3. Specifically, PASI 90 was achieved by 53%, 72%, 73%, and 82% at weeks 16, 28, 40, and 52, respectively, this outcome at 16 weeks is slightly lower than ones reported in the phase III studies UltIMMa‐1 (75.3%), UltIMMa‐2 (74.8%),^3^ IMMerge (73.8%),^5^ SustaIMM (75%)^7^ and IMMvent (72%).^6^

73% of patients reached PASI 75 at week 16, which was lower than in the SustaIMM (90 and 95%), IMMerge (92%), and IMMvent (91%) studies. PASI 100 was achieved at week 16 in our population by the 32% of the cohort, also slightly lower than UltIMMa‐1 and 2 (35.9% and 50.7%, respectively), SustaIMM (22 and 33%), IMMerge (44%) and IMMvent (50%). Compared to the UltIMMA‐1 and ‐2 studies at 52 weeks, the percentage of patients achieving PASI 100 and 90 in our population was slightly higher (56.3% and 50.7% vs. 73% for PASI 100; 81.9% and 80.6% vs. 82% for PASI 90). The percentage of patients reaching PASI 100, 90 and 75 at 52 weeks in our study was similar to IMMerge (73% vs. 66%, 82% vs. 87%, 91% vs. 90%, respectively). Our data, although limited by the low sample size, would appear to show a slower rate of action of the drug than in the registration studies, while maintaining a similar if not superior response over the long term. Significant differences are usually found in real‐life studies compared to registration studies due to different patient demographics. Our population is older and more bio‐experienced than IMMerge, UltIMMa‐1 and ‐2 population (65.1 years vs. 47.3, 48.3, 46.3 and 58% vs. 37.8%, 34%, 40%, respectively). The high number of failures with previous interleukin 17 and 23 inhibitor therapy, as well as a high proportion of multi‐failure (7%) compared to currently available studies, may have resulted in a slower response than reported in these studies. The mean initial PASI of 12.5 of our population is lower than the starting PASI of the registry studies (e.g. UltIMMA1 and 2 20.6 and 20.5), which could reduce the relative response for the weeks considered. On the other hand, in our population, PsA affects fewer patients than in the registration studies (13% vs. 28% and 25% of UltIMMA‐1 and ‐2), and the mean BMI is also lower (26.7 vs. 29.9 and 31.1). Joint involvement and high weight are well‐known unfavorable factors for treatment response, so our patients compared to the phase III studies should have experienced less impact for these factors on PASI outcomes. However, we cannot forget that PASI remains an operator‐dependent assessment and even appreciable variations are shown between different operators, in particular between studies with different methodological rigor e.g. between retrospective studies such as ours and phase III studies.

Real‐life studies on the efficacy of risankizumab are still scarce, and the sample size is small. Our study, together with the Czech study, has the largest starting population and longest follow‐up.^2^ Even in comparison with that study, the outcomes observed in our population were lower in the achievement of PASI 100 and PASI 90 at weeks 16 (32% vs. 44.7% and 53% vs. 63.8%), and weeks 28 (51% vs. 59.1% and 72% vs. 77.3%) and then reached and exceeded the response at week 52 (73% vs. 67.7% and 82% vs. 82.4%). Compared to the Italian study by Hansel et al., outcomes at 16 weeks for PASI 75, 90, and 100 are also lower (73% vs. 86%, 53% vs. 63.2, and 32% vs. 49.1%).^9^ Similarly, our results were lower than those of the recent study by Borroni et al. in reaching PASI 75, PASI 90, and PASI <3 but not in reaching PASI 100 (73% vs. 85.7%, 53% vs. 61%, and 32% vs. 28.7%). The same study reported the largest real‐life case series at 40 weeks, with results superior to ours: 98.7% 85.7% 96.1% and 62.3% of patients achieved PASI 75, 90, <3, and 100, respectively, compared with 83%, 73%, 87%, and 53% of our patients.^15^ Compared to the Czech study we started with a lower mean PASI at baseline (16.7 vs. 12.5) and the mean age was lower (48.5), however the BMI, obese, bio‐experienced, and PsA presence data reported in these studies are higher or comparable to ours.

To identify possible predictors of response to treatment, we compared various subpopulations of patients with each other. In agreement with previous studies, we compared patients based on their previous‐biologic status and BMI, in particular the obese versus non‐obese population (BMI <30). We also analyzed concerning the habit of smoking which, as mentioned, is a recognized disadvantage response factor to treatments in the psoriatic patient, compared patients with difficult/special sites involved to non‐difficult, and concerning the presence or absence of PsA.

Significant differences were found between bio‐naive and bio‐experienced patients (42% and 58%, respectively), in particular in the achievement of PASI 90 and PASI 75 at weeks 16 and 28. This data is partially in contrast with current real‐life literature,^2^, ^9^the study by Hansel et al. identifies a significant difference in the attainment of PASI 100 at weeks 36 and 52, Borroni et al. described mild difference in the achievement of PASI 75 at week 16, and relevant difference at week 40. In any case, the bio‐experienced patients in our population show a progressive improvement in response over time, at 40 weeks differences compared to the bio‐naive were detected only in the achievement of PASI 90, however with low statistical significance (64% vs. 100%, *p* = 0.046). In our cohort the percentage of bio‐experienced patients is higher than in the UltIMMA‐1 and ‐2 studies, but slightly lower than in the Italian and Czech studies.^2^, ^3^, ^4^, ^8^, ^9^, ^10^ In particular, the number of patients who had previously taken another inhibitory therapy for IL‐23, such as Guselkumab and Tildrakizumab, is higher than all cases reported in the literature (2 patients in real life and 3 patients in phase III studies), for a total of 10 patients (5.6%).^5^, ^10^ In conclusion, a previous history of using biological drugs may reduce response to risankizumab.

The scalp, folds, palms, nails, and genitals have traditionally been considered difficult sites in the treatment of psoriasis due to the reduced efficacy or difficult application of topical treatment. Systemic treatment including biological treatment should lead to better results in these sites.^11^ Hjuler et al. in an observational study found that sites traditionally considered difficult responded well to biologic therapy, except for the scalp, identifying the lower legs and elbow as the most resistant sites.^11^ In our study a high percentage of patients had involvement of difficult sites (73%) compared to the registration and other real‐life studies, however, not all sites we considered as difficult were evaluated as such by other studies. In our population, there was no substantially difference between the population with at least one of the difficult sites involved and the population without the involvement of these, only in the achievement of PASI 90 at week 16 we observed differences (47% vs. 69%, *p* = 0.012). In two studies, Megna et al. showed that the scalp was the site with the best response to risankizumab, followed by the plantar‐palm area; only the nail area showed a slower improvement, without significant differences between each site.^3^, ^10^ These results, in line with those shown by the UltIMMA‐1 and ‐2 and IMMhance studies,^2^, ^4^ describe Risankizumab as highly effective on difficult sites in both trials and real life settings.

A high BMI is a well‐known negative predictor of psoriasis response to some biologic treatment as anti‐TNFalpha, ustekinumab and secukinumab.^16^, ^17^, ^18^ High weight does not seem to compromise the response to brodalumab,^19^ the role of the weight in the response to ixekizumab shows contradictory results in the current literature.^18^, ^20^ In our study obesity proved to be a negative predictor of response to risankizumab in some of the outcomes analyzed: in the achievement of PASI 100 at week 28, reached by 22% of obese patients versus 58% of non‐obese patients (*p* = 0.007), and in the mean PASI at 28 weeks 1.7 vs. 0.9 (*p* = 0.006). Other significant differences were seen in the attainment of PASI 100 at week 40 and PASI <3 at week 40. Our data seem to partly contradict the results reported by Gkalpakiotis et al. that found no significant differences in patients with higher BMI and to be more in line with the data of Hansel et al. that show a lower response in the overweight or obese population.^2^, ^8^ The comparison is, however, difficult considering the different levels of BMI analyzed between our study and the other real‐life studies (≥30 BMI vs. <30 in our study, <25 vs. ≥25 in the cited studies).

Although the smoking habit is considered a negative factor in the course of psoriasis and its response to biologics, as recently mentioned in the systematic review by Zhou et al., the response to risankizumab has never been evaluated concerning presence or absence of this habit, neither in phase III clinical trials nor in real‐life studies.^2^, ^3^, ^12^ In our study we found limited differences attributed to smoking habits. We subdivided the population into current, past, and absent smoking habits, and found significant differences in favor of absent smoking habits, both in the reduction of mean PASI (*p* = 0.026), and in the achievement of the relative PASI 100 at 40 weeks (*p* = 0.029). This difference is, however, less consistent than in the obese or bio‐experienced population.

According to the latest European guidelines, anti‐TNFalpha together with ixekizumab and secukinumab are the drug of choice in the second‐line treatment of psoriatic arthritis after methotrexate.^21^ Recently brodalumab, guselkumab and tildrakizumab have demonstrated safety and efficacy in patients with this type of disease.^22^, ^23^, ^24^ There is currently no study demonstrating the superiority of one biologic over another for the treatment of PsA. Existing studies have shown no superiority of anti‐IL17 over anti‐TNFalpha in the treatment of PsA, in contrast to the clear superiority of anti‐IL‐23 and IL‐17 in the resolution of skin involvement. Recent advances in psoriasis therapy have increased the therapeutic possibilities of PsA, although only a partial response is observed in about 40% of patients, underlining the different pathological substrate of this disease compared to the cutaneous version.^14^ To date, there is no study dedicated to risankizumab in PsA patients. Although in phase III and real‐life studies the affected population also at the joint level is between 20–30%, no specific efficacy analysis has been performed on this subpopulation.^2^, ^3^, ^4^ In our study, 22% of patients had joint involvement, which is in line with the current literature, and showed a generally poorer response to treatment. Significant differences in mean PASI at any time‐point was observed between PSA and non‐PSA patients: 2.7 versus 1.7 (*p* = 0.036), 1.9 versus 0.9 (*p* = 0.006) and 4.1 versus 0.5 (*p* = 0.016) at 16, 28, 40 weeks in PSA, respectively. Other significant differences were found in the attainment of the PASI 100, PASI 90, PASI 75 and PASI <3 at any endpoints. Based on these data, we can assume a lower efficacy of risankizumab in psoriatic arthritis.

The safety of risankizumab over 52 weeks was good, with only one side effect‐related discontinuation (0.6% of the total population), a result similar to that reported by phase III and same nature studies.^2^, ^4^ No new safety signals were observed. These results were recently underlined by a study on the safety of risankizumab and other anti IL23 in frail, elderly patients with numerous comorbidities.^25^

In our population, one event of peri‐malleolar edema leading to treatment discontinuation occurred, this event was not previously reported. One patient died before week 16. Death was correlated to the patient's previous general condition and not to the treatment. No recurrence or new neoplasia were observed. Only 8 patients (4.8%) stopped the treatment, mainly due to primary inefficacy. Indeed, adverse events led to treatment discontinuation in only 1 patients (0.6%), further supporting risankizumab high safety profile.

Our study shares the usual limitations of real‐life studies, retrospective design, and absence of a control group. The number of patients is relatively low, although higher than the real‐life studies currently present in the literature. The sample, also tend to reduce during the following time‐points analyzed, making it impossible to perform statistical analysis at week 52.

## 7 CONCLUSIONS

In our study, risankizumab showed efficacy and optimal safety profile. Our data support what was previously highlighted by clinical trials and previous real‐life studies, addressing outcomes not analyzed in previous studies such as PASI <3. The efficacy was slightly slower than that reported in trials at all the outcomes analyzed, with PASI outcomes being inferior at week 16 but equaling the data of clinical trials and other real‐life at later time‐points. The high operator‐dependence of PASI as an efficacy measure may have critically plagued our results.

This slow and progressively greater effect could be associated with a persisting response over time, and longer studies are needed to confirm this hypothesis. The lower number of administrations required by Risankizumab is not to be overlooked in those patients whose extent of disease does not justify repeated and close administration but whose burden of disease makes biological treatment necessary.

Our data show that obesity, previous biologic experience and above all joint involvement determine a lower response to treatment. Less pronounced differences were found with respect to smoking habits and difficult site involvement in our study only affects PASI 90 outcome at 16 weeks, generally we can say that Risankizumab did not show different among smoking habits, and classic and difficult sites. Real‐life studies with higher numbers especially at higher time‐points and with longer follow‐up could better estimate the efficacy of risankizumab in the clinical setting. Proof‐of‐concept studies are needed to better evaluate the weight of possible prognostic factors in the psoriasis response to risankizumab.

## CONFLICT OF INTEREST

The authors report no conflict of interest.

## AUTHOR CONTRIBUTIONS

Luca Mastorino, Sara Susca, Matteo Megna, Niccolò Siliquini, Pietro Quaglino, Michela Ortoncelli, Gianluca Avallone, Marco Rubatto, Gabriella Fabbrocini, Paolo Dapavo, and Simone Ribero participated in the drafting and revision of the work. Luca Mastorino, Sara Susca, Matteo Megna, Niccolò Siliquini, Pietro Quaglino, Michela Ortoncelli, Gianluca Avallone, Marco Rubatto, Gabriella Fabbrocini, Paolo Dapavo, and Simone Ribero read and approved the manuscripts and give consent for publication.

## ETHICS STATEMENT

The study was approved by the ethics committee of Turin University hospital (IT10771180014 SS‐Dermo20). All the participants read and signed informed consent.

## Data Availability

Data available upon reasonable request.
